# A Multicenter Study Validates the WHO 2022 Classification for Conjunctival Melanocytic Intraepithelial Lesions With Clinical and Prognostic Relevance

**DOI:** 10.1016/j.labinv.2023.100281

**Published:** 2023-11-03

**Authors:** Hardeep Singh Mudhar, Yamini Krishna, Simon Cross, Claudia Auw-Haedrich, Raymond Barnhill, Svetlana Cherepanoff, Ralph Eagle, James Farmer, Robert Folberg, Hans Grossniklaus, Martina C. Herwig-Carl, Martin Hyrcza, Sandra Lassalle, Karin U. Loeffler, Alexandre Moulin, Tatyana Milman, Robert M. Verdijk, Steffen Heegaard, Sarah E. Coupland

**Affiliations:** a National Specialist Ophthalmic Pathology Service, Department of Histopathology, E-Floor, Royal Hallamshire Hospital, Sheffield, UK; b National Specialist Ophthalmic Pathology Service, Liverpool Clinical Laboratories, Liverpool University Hospitals NHS Foundation Trust, Liverpool, UK; c Liverpool Ocular Oncology Research Group, Department of Molecular and Clinical Cancer Medicine, Institute of System Molecular and Integrative Biology, University of Liverpool, Liverpool, UK; d Academic Unit of Pathology, Department of Neuroscience, University of Sheffield, Sheffield, UK; e Eye Centre, Faculty of Medicine, University of Freiburg, Freiburg, Germany; f Department of Translational Research, Institut Curie, Paris Sciences and Lettres Research University, and Faculty of Medicine University of Paris Descartes, Paris, France; g Sydpath, Department of Anatomical Pathology, St Vincent’s Hospital, Sydney, New South Wales, Australia; h Faculty of Medicine, University of New South Wales, Sydney, New South Wales, Australia; i Department of Pathology, Wills Eye Hospital, Sidney Kimmel Medical College of Thomas Jefferson University, Philadelphia, Pennsylvania; j Department of Ophthalmology, Wills Eye Hospital, Sidney Kimmel Medical College of Thomas Jefferson University, Philadelphia, Pennsylvania; k Departments of Ophthalmology and Pathology and Molecular Medicine, Queen’s University, Kingston, Ontario, Canada; l Departments of Pathology and Laboratory Medicine and Ophthalmology, University of Ottawa, Ottawa, Ontario, Canada; m Departments of Ophthalmology and Pathology, Oakland University William Beaumont School of Medicine, Rochester, Michigan; n Departments of Ophthalmology and Pathology, Corewell Health William Beaumont University Hospital, Royal Oak, Michigan; o Department of Ophthalmology, Ocular Oncology and Pathology Section, Emory Eye Center, Emory University School of Medicine, Atlanta, Georgia; p Department of Ophthalmology, Division of Ophthalmic Pathology, University Hospital Bonn, Bonn, Germany; q Department of Pathology and Laboratory Medicine, University of Calgary, Arnie Charbonneau Cancer Institute, Calgary, Alberta, Canada; r Laboratory of Clinical and Experimental Pathology, Hospital-Related Biobank (BB-0033-00025), Pasteur Hospital, Centre Hospitalier Universitaire de Nice and Institute of Research on Cancer and Aging, FHU OncoAge, Université Côte d’Azur, Nice, France; s Jules-Gonin Eye Hospital, Fondation Asile des Aveugles, University of Lausanne, Lausanne, Switzerland; t Department of Pathology, Section of Ophthalmic Pathology, Erasmus MC University Medical Center Rotterdam, Rotterdam, The Netherlands; u Department of Pathology, Leiden University Medical Center, Leiden, The Netherlands; v Department of Pathology, Eye Pathology Section, and Ophthalmology, Rigshospitalet, University of Copenhagen, Denmark

**Keywords:** conjunctival melanoma, digital pathology, high-grade conjunctival melanocytic, intraepithelial lesions, immunohistochemistry, low-grade conjunctival melanocytic, intraepithelial lesions, conjunctival melanoma in situ

## Abstract

Several nomenclature and grading systems have been proposed for conjunctival melanocytic intraepithelial lesions (C-MIL). The fourth “WHO Classification of Eye Tumors” (WHO-EYE04) proposed a C-MIL classification, capturing the progression of noninvasive neoplastic melanocytes from low- to high-grade lesions, onto melanoma in situ (MIS), and then to invasive melanoma. This proposal was revised to the WHO-EYE05 C-MIL system, which simplified the high-grade C-MIL, whereby MIS was subsumed into high-grade C-MIL. Our aim was to validate the WHO-EYE05 C-MIL system using digitized images of C-MIL, stained with hematoxylin and eosin and immuno-histochemistry. However, C-MIL cases were retrieved from 3 supraregional ocular pathology centers. Adequate conjunctival biopsies were stained with hematoxylin and eosin, Melan-A, SOX10, and PReferentially expressed Antigen in Melanoma. Digitized slides were uploaded on the SmartZoom platform and independently scored by 4 ocular pathologists to obtain a consensus score, before circulating to 14 expert eye pathologists for independent scoring. In total, 105 cases from 97 patients were evaluated. The initial consensus diagnoses using the WHO-EYE04 C-MIL system were as follows: 28 benign conjunctival melanoses, 13 low-grade C-MIL, 37 high-grade C-MIL, and 27 conjunctival MIS. Using this system resulted in 93% of the pathologists showing only fair-to-moderate agreement (kappa statistic) with the consensus score. The WHO-EYE05 C-MIL system (with high-grade C-MIL and MIS combined) improved consistency between pathologists, with the greatest level of agreement being seen with benign melanosis (74.5%) and high-grade C-MIL (85.4%). Lowest agreements remained between pathologists for low-grade C-MIL (38.7%). Regarding WHO-EYE05 C-MIL scoring and clinical outcomes, local recurrences of noninvasive lesions developed in 8% and 34% of the low- and high-grade cases. Invasive melanoma only occurred in 47% of the cases that were assessed as high-grade C-MIL. This extensive international collaborative study is the first to undertake a comprehensive review of the WHO-EYE05 C-MIL scoring system, which showed good interobserver agreement and reproducibility.

## Introduction

Correct classification of melanocytic lesions of the conjunctiva is key for the treatment of patients with both preinvasive and invasive diseases. Several terminology and classification systems have been proposed over the decades for the preinvasive staged—ie, conjunctival intraepithelial melanocytic proliferations—each with its strengths and weaknesses. The most used in diagnostic practice to date include the primary acquired melanosis (PAM) with atypia system (PAM),^1^ the conjunctival melanocytic intraepithelial neoplasia (C-MIN) system (C-MIN),^2^ and the intraepithelial melanocytic proliferation system.^3^

In 2018, the editorial board of the fourth “WHO Classification of Eye Tumors” puts forward a proposal for a simplified classification scheme for conjunctival melanocytic intraepithelial lesions (C-MIL).^4^ This WHO-EYE04 system comprised the following: low-grade C-MIL, high-grade C-MIL, and melanoma in situ (MIS). Benign conjunctival melanocytic lesions, including hypermelanosis of the basal conjunctival epithelial layer and racial melanosis, were not included in this system. In 2021, Milman et al^5^ performed a validation of the WHO-EYE04 C-MIL system in 64 patients and compared its performance with the PAM with atypia and the C-MIN classifications. The interobserver agreement, based on hematoxylin and eosin (H&E)-stained sections only, for the distinction between the low- and high-grade C-MIL was 76% for the “PAM with atypia” system, 67% for the C-MIN system, and 81% for the WHO-EYE04 system. These 3 classification systems had a comparable accuracy of 81%–83% in their ability to identify lesions with potential for recurrence. Two weaknesses of the Milman study^5^ were that it did not include benign melanocytic lesions for evaluation (although it did put forward a modified WHO-EYE04 C-MIL system for their inclusion) and included only 2 cases that progressed to melanoma. Hence, regarding the latter, this study could not provide information regarding how predictive the classification system was for melanoma progression.

**Figure 1.**
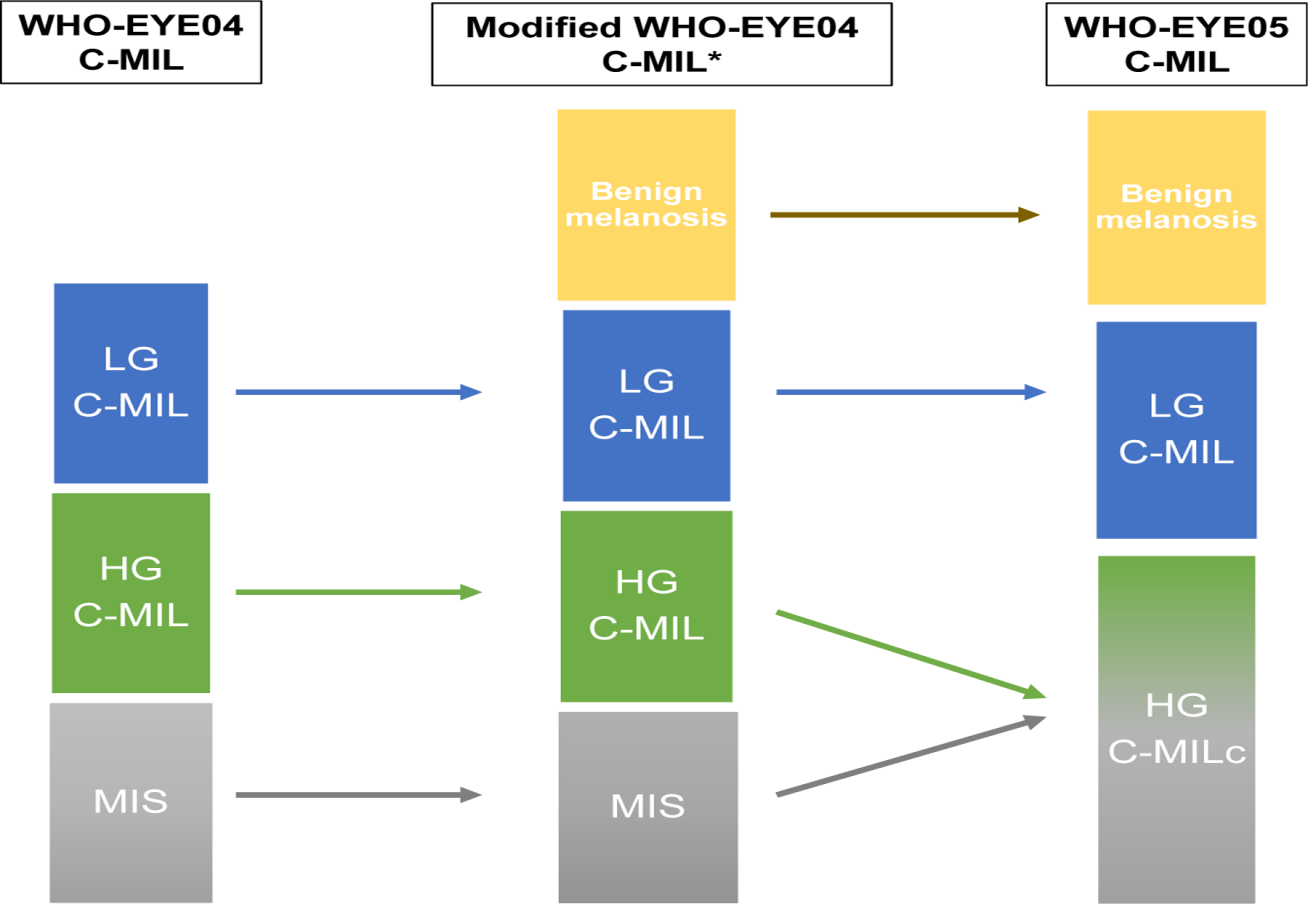
Pictorial flow diagram showing the evolution of the WHO-EYE04 and WHO-EYE05 C-MIL grading systems. The modified WHO-EYE04* was proposed in the Milman et al^5^ study but revised further by the Editorial Board of the WHO-EYE05 into the WHO-EYE05 system. LG C-MIL, low-grade conjunctival melanocytic intraepithelial lesion; HG C-MIL, high-grade conjunctival melanocytic intraepithelial lesion; MIS, melanoma in situ melanoma; HG C-MIL, c high-grade conjunctival melanocytic intraepithelial lesion and MIS combined.

In 2022, the fifth “WHO Classification of Eye Tumors” (WHO-EYE05) revised the C-MIL system slightly further to include the benign melanoses (equating to C-MIN score 0–1 and PAM without atypia) but created only 2 neoplastic groups, namely low-grade C-MIL (equating to C-MIN scores 2–4 and PAM with mild atypia) and high-grade C-MIL (equating to PAM with moderate and severe atypia and MIS, and a C-MIN score of 5–10; Fig. 1 and Table 1).^6^ The motivation behind this revision was that the system should reflect the biology of the lesion, be predictive of recurrence and invasive disease, and reflect clinical treatment thresholds.

In the present study, we evaluated the modified versions of the WHO-EYE04 and WHO-EYE05 C-MIL scoring systems on a cohort of 105 cases from 97 patients with clinical follow-up, as assessed by 18 experienced pathologists from the UK, Europe, North America, and Australia. This multicenter study, which included benign melanoses and cases that had progressed to melanoma, was undertaken using a digital platform, where each case had a H&E section with accompanying Melan-A, SOX10, and PRAME (PReferentially expressed Antigen of MElanoma) immunostains and associated clinical information, simulating the usual diagnostic scenario. This analysis was performed to assess and attempt to ensure reproducibility between pathologists in scoring these difficult lesions, not as an academic exercise but rather to seek a consensus on when to “flag” to clinicians that active treatment of the patients would be required versus observation alone.

## Materials and Methods

The Liverpool University Hospitals NHS Foundation Trust approved and hosted this international collaborative registered audit. The study was performed in compliance with the tenets of the Declaration of Helsinki.

### Patient and Tissue Selection

The histopathological data archives were searched for C-MIL cases, including benign melanoses and cases that had ultimately progressed to melanoma, in the following 3 ocular oncology/pathology centers: Liverpool University Hospitals NHS Foundation Trust (Liverpool; cases from 2018 to 2021), Royal Hallamshire Hospital (Sheffield; from 2011 to 2021), and Rigshospitalet (Copenhagen; from 1996 to 2021). Patients had undergone primary surgery for “conjunctival melanosis” in the Ocular Oncology Services of each of the respective hospitals. All surgeries were performed using standard techniques, and all patients had postoperative follow-up data for at least 36 months. The histopathology slides of each case were reviewed for adequacy by each of the respective centers and again by the host center, before inclusion in the study. Biopsies that were suboptimal for histopathologic interpretation were excluded.

Collected clinical data included the following: patient age at the time of surgery, sex, ethnicity, tumor laterality, anatomical location of conjunctival lesion, clinical follow-up period (all cases were followed up by at least 36 months), number of recurrences, presence/absence of invasive melanoma, presence/absence of metastases, and cause of death (if appropriate).

### Histopathologic Evaluation

Glass slides of each case were routinely prepared from the formalin-fixed paraffin-embedded conjunctival biopsies. Tissues were stained with H&E and immunohistochemistry (IHC) with the following antibodies: Melan-A (clone A103; dilution1:50; Agilent Dako; Dako Omnis Platform), SOX10 (clone EP268; dilution1:400; Cell Marque; Dako Omnis Platform), and PRAME (clone QR005; ready to use; Anatopath; BenchMark Ultra Platform). These were applied as per manufacturer’s protocols and ISO15189 validation.

Although all 3 immunostains had not been performed, any missing stains were undertaken in Liverpool by the NHS Liverpool Clinical Laboratories. Once all immunostains for each case were present, the pseudonymized slides of all cases were scanned and digitized using the VENTANA DP 200 slide scanner (Roche Diagnostics International AG) at 40× magnification and uploaded and displayed on the Smart Zoom platform (Smart In Media AG; available at https://www.smartinmedia.com/smartzoom-classroom/).^7^ Although all digitized slides did not have any identifiable clinical data on them, basic information for each respective case was displayed on the SmartZoom platform and included the following: patient age at the time of surgery, sex, ethnicity, tumor laterality, and anatomical location.

**Figure 2.**
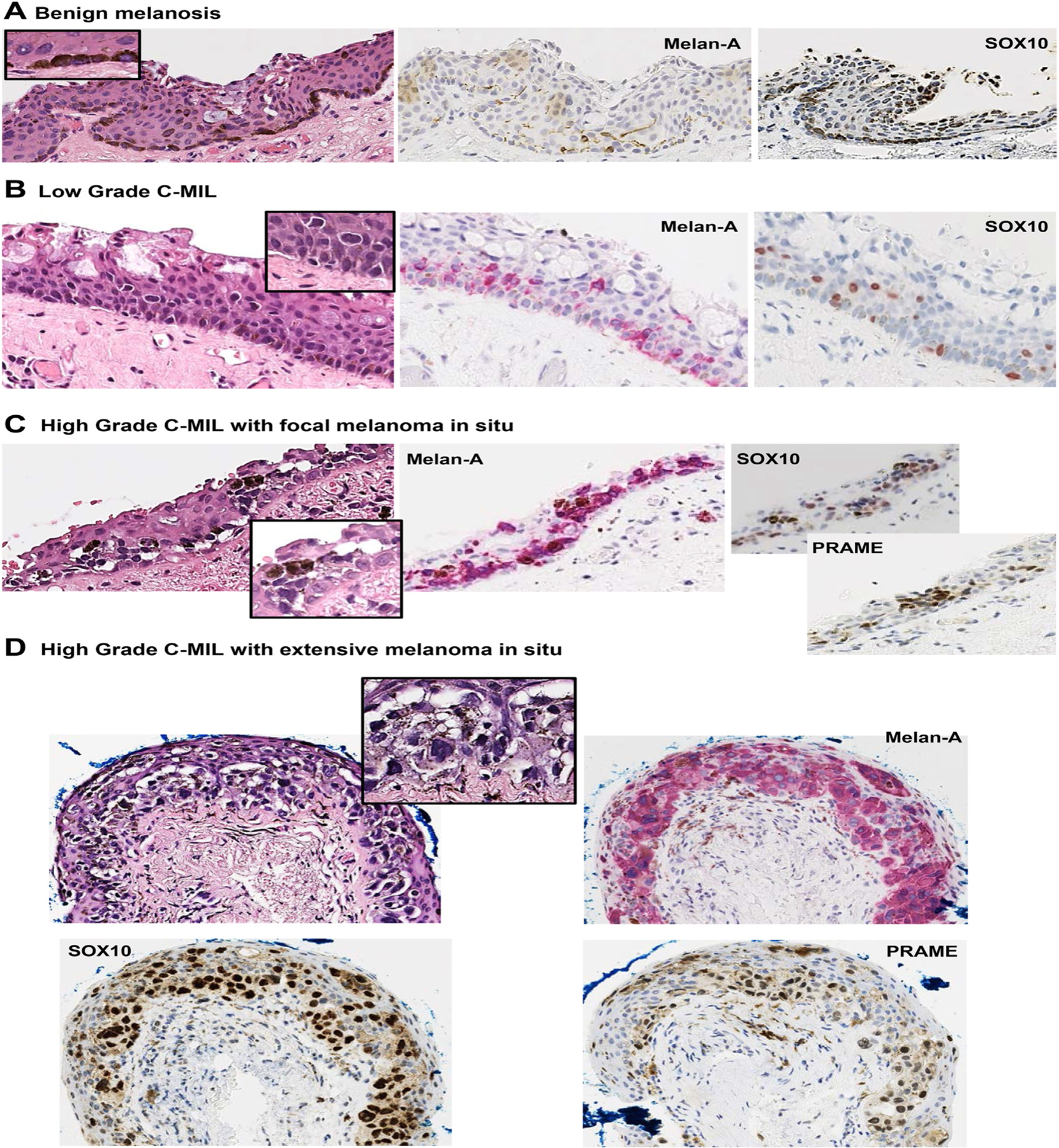
Photomicrographs showing the H&E section and corresponding immunohistochemistry for each of the C-MIL scoring grades. C-MIL, conjunctival melanocytic intraepithelial lesion.

The pseudonymized digitalized slides for each case (H&E, Melan-A, SOX10, and PRAME) were initially scored independently and in a masked manner (ie, without knowledge of clinical outcome) by the 4 observers from each of the 3 study centers (Y.K. and S.E.C. [Liverpool], H.S.M. [Sheffield], and S.H. [Copenhagen]). The cases, which included benign melanoses and cases that had progressed to melanoma, were scored using modified versions of WHO-EYE04 (4-tiered) and WHO-EYE05 (3-tiered) systems for C-MIL [see Fig. 1 and Table 1 for the histomorpho-logical descriptions of each lesional grade].^6^ Briefly, the 4-tiered scoring system comprised the following categories: 1 = benign melanosis, 2 = low-grade C-MIL, 3 = high-grade C-MIL, and 4 = frank MIS (Fig. 2). The WHO-EYE05 (3-tiered) C-MIL system comprised only of the following: 1 = benign melanosis, 2 = low-grade C-MIL, and 3 = high-grade C-MIL, including MIS. The highest grade of C-MIL present in the lesion was recorded per case in a spreadsheet.

Parameters noted per case by the pathologists included the following: the most useful IHC stain in decision-making and the presence/absence of a naevus-, stromal microinvasion-, and frank-invasive melanoma. Any discrepant scores between the 4 initial observers (Y.K., S.E.C., H.S.M., and S.H.) were rereviewed at a consensus meeting before the final consensus score was achieved. The final consensus score established for each case was used for data analyses.

Access to these 105 digitalized cases on the SmartZoom platform was provided to 14 experienced ocular pathologists from Europe, North America, and Australia (co-authors were as follows: C.A-H., R.B., S.C., R.E., J.F., R.F., H.G., M.H-C., M.H., S.L., K.L., A.M., T.M., and R.V.), who then independently scored them using the WHO-EYE05 classification system for C-MIL. Eleven of the pathologists involved in this current study also participated in the previous analysis by Milman et al;^5^ however, it is important to note that 6 additional judging pathologists were also included.

All scoresheets were returned to Y.K., and the results were anonymously collated for independent statistical analysis by co-author S.C.R. (See Supplementary Fig. S1 for the workflow of the methods.)

### Statistical Analysis

The kappa statistics were calculated using the Fleiss method in R^8^ (The R Project for Statistical Computing^9^) with the Epi package^10^ between the consensus score evaluated by the study co-ordinators (Y.K., S.E.C., H.S.M., and S.H.) and the 14 above-mentioned ophthalmic pathologists. Kappa scores were indicated as the following: <0.2 = slight agreement, 0.2–0.4 = fair agreement, 0.4–0.6 = moderate agreement, 0.6–0.8 = substantial agreement, and >0.8 corresponded to almost perfect agreement.^8^

## Results

### Basic Demographic Data of the Submitted Cases

In total, a cohort of 105 C-MIL cases from 97 patients were evaluated. This cohort comprised of 60 women (age range: 23–93 years; median 65; mean 61.5) and 37 men (age range: 31–91 years; median 68; mean 66.5). The ethnic group mix comprised the following: 88 White Caucasian, 4 Black, 2 South Asian, 1 Inuit, 1 Mixed race, and 1 Unspecified. The mean follow-up data varied between the ocular oncology centers at Copenhagen, Liverpool, and Sheffield with the mean being 169, 42, and 77 months, respectively.

The ocular laterality of the lesions was as follows: 50 left eyes, 47 right, and 8 unspecified. The exact locations of the lesions were as follows: 48 bulbar, 26 limbal, 9 fornix, 6 plica, 4 limbal and bulbar, 3 palpebral, 2 caruncle, and 7 unspecified.

### Basic Diagnostic and Clinical Data

According to the initial consensus panel, the conjunctival melanocytic lesions were classified using the modified WHO-EYE04 as the following: 28 benign melanosis, 13 low-grade C-MIL, 37 high-grade C-MIL, and 27 conjunctival MIS. The local recurrence rate was as follows: no recurrence in 81/105 cases (77%), recurrence in 23/105 cases (22%), and in 1 case, data were not available. In 74/105 (70%) cases, no invasive melanoma was observed during the disease. In 30/105 (29%) cases, invasive melanoma developed, and in 1/105 (1%), data were not available.

### Statistical Analysis

#### Straight Comparison of All Cases Between Consensus Panel and Participating Pathologists

Supplementary Table S1 shows the kappa statistics with 95% CI, for the 14 participating pathologists scoring the 105 cases, compared with the consensus diagnosis, using this modified 4-tiered WHO-EYE04 C-MIL grading system of benign melanosis, low-grade C-MIL, high-grade C-MIL, and MIS. In summary, 5 observers produced fair agreement with the consensus score, 8 moderate agreements, but only one observer with substantial agreement.

When the results were re-evaluated using the 3-tiered WHO-EYE05 C-MIL system (whereby the high-grade C-MIL and MIS categories were combined), the concordance between pathologists improved. Table 2 shows the kappa statistics with 95% CI, for the 14 participating pathologists grading the 105 cases, compared with the consensus diagnosis, using the WHO-EYE05 C-MIL system. Briefly, only one observer showed fair agreement, 6 showed moderate agreement, and 7 showed substantial agreement.

Scrutiny of these 2 tables showed an increase in the kappa statistic between the 4- and 3-grade systems, which was statistically significant at a 5% level for 10 of the 14 observers.

#### Agreement Comparison for the Various Conjunctival Melanocytic Intraepithelial Lesions Grades

We evaluated the level of agreement between the consensus panel and 14 participating pathologists for specific diagnostic categories within the 4-tiered WHO-EYE04- and 3-tiered WHO-EYE05 C-MIL systems (Table 3).

For the 4-tiered WHO-EYE04 C-MIL system, the highest level of agreement was seen with benign melanosis (74.5%) and the lowest for low-grade C-MIL (38.7%). Using WHO-EYE05 C-MIL grading, the highest level of agreement was for high-grade C-MIL (85.4%) and the lowest with low-grade C-MIL (38.7%).

#### Grade of Conjunctival Melanocytic Intraepithelial Lesions and Relationship to Recurrence and Development of Invasive Melanoma

For the 4-tiered WHO-EYE04 C-MIL, local recurrence occurred in 0% benign melanosis, 8% low-grade C-MIL, 18% high-grade C-MIL, and 55% MIS cases (Supplementary Table S2). Development of invasive melanoma was seen in only the high-grade C-MIL and in situ melanoma cases in 27% and 74% of the cases, respectively (Supplementary Table S2).

For the 3-tiered WHO-EYE05 C-MIL grading, local recurrence of noninvasive disease was seen in benign melanosis, low-grade C-MIL, and high-grade C-MIL in 0%, 8%, and 34% of the cases, respectively (Table 4). Invasive melanoma subsequently developed in 47% of the high-grade C-MIL cases only; none was observed in cases classified as benign melanosis or low-grade C-MIL (Table 4).

#### Immunostain Preference Among Participating Pathologists for Conjunctival Melanocytic Intraepithelial Lesions Grades That Agreed With the Consensus Grade)

In 84% of all participating pathologists’ responses, the IHC preference was recorded against the grade of C-MIL (Supplementary Table S3A). When considering the cases where there was agreement on the C-MIL grade between the initial consensus panel and all participating pathologists, SOX10 and Melan-A (or a combination of both) were the most preferable immunostains across all grades of C-MIL.

When examining individual markers, SOX10 was most frequently employed for benign melanosis and less frequently for MIS. Comments by pathologists suggested that SOX10 nuclear expression allowed for the assessment of nuclear size, shape, and pattern. Melan-A was most useful for high-grade C-MIL, allowing for assessment of the vertical thickness of the lesion within the epithelium. However, PRAME was used, individually or in combination with other antibodies for assessing high-grade C-MIL and MIS. Therefore, PRAME was absent in low-grade lesions but present in higher-grade lesions, including MIS.

Differing combinations of the 2 IHC stains were found to help achieve the following diagnoses: 22% of benign melanosis, 28% of low-grade C-MIL, 27% of high-grade C-MIL, 35% of MIS, and 30% of high-grade C-MIL (combined), of which the commonest combination was Melan-A and SOX10. All 3 antibodies were employed for 4% of benign melanosis, 5% of low-grade C-MIL, 4% of high-grade C-MIL, 20% MIS, and 12% of high-grade C-MIL (combined).

#### Immunostain Preference Among Participating Pathologists for Conjunctival Melanocytic Intraepithelial Lesions Grades at Variance With the Consensus Grade (Supplementary Table S3B)

We also wanted to gain some insights into the pattern of IHC usage when the participating pathologists’ C-MIL grades were at variance with the consensus diagnosis. As seen in Supplementary Table S3B, Melan-A and SOX10 were the most utilized, singly or in combination, across all grades. Combinations of 2 or 3 immunostains were more commonly employed with higher-grade lesions. Therefore, PRAME was used more frequently for high-grade C-MIL, high-grade C-MIL (combined), and MIS, singly or in combination.

## Discussion

This large international collaborative study is the first to undertake a detailed comprehensive review of C-MIL, using digital pathology and a routine panel of conventional H&E and IHC, to validate the WHO-EYE05 system for these lesions. An analysis of 105 cases, sourced from 3 different ocular oncology/ophthalmic pathology centers and scored independently by 18 histopathologists, was conducted to determine the reproducibility of C-MIL scoring using a modified 4-tiered WHO-EYE04 system and the proposed WHO-EYE05 system, which encompasses 3-tiers only. The latter WHO-EYE05 C-MIL system was found to have substantial agreement between observers, be the most reproducible, and be concordant with clinical outcomes. We would suggest that this is the system to move forward with in diagnostic laboratories.

The nomenclature of conjunctival melanocytic lesions has been fraught with problems and indeed battles over the decades.^2,11^ In 2018, the WHO-EYE04 proposed a classification scheme for C-MIL, comprising low-grade C-MIL, high-grade C-MIL, and MIS.^4^ The aims of this new classification were as follows: (1) to use terms that reflected the underlying biology of the lesions, (2) to simplify their grading, yet still accurately capture their risk of disease progression (prognostic accuracy) compared with the existing PAM and C-MIN systems, and (3) to facilitate decision-making for the treating clinicians as to which C-MIL groups could be observed/“watch and wait” versus the groups that required more aggressive treatment (surgery, brachytherapy, and/or topical chemotherapy, eg, mitomycin C).

A multicenter study validated this WHO-EYE04 C-MIL system and compared its performance with the “PAM with atypia” and “C-MIN” classification systems.^5^ The interobserver agreement when discerning between the low- and high-grade lesions was 76% for PAM with atypia systems, 67% for C-MIN systems, and 81% for the newly proposed WHO classification system. The 3 classification systems had a comparable accuracy of 81%–83% in their ability to identify lesions with potential for recurrence. Therefore, the WHO-EYE04 C-MIL system was considered to be “user friendly” that would allow for the easier grading of conjunctival melanocytic lesions and flag to clinicians which patients would benefit from postoperative treatment to hinder disease progression.

However, it remained apparent that the WHO-EYE04 C-MIL system had 2 main weaknesses—namely, that it did not include the most common benign conjunctival melanocytic lesions: (a) those caused by hypermelanosis of the epithelial layers of the conjunctiva or (b) racial melanosis. Furthermore, the definition distinguishing high-grade C-MIL and MIS remained arbitrary, with the percentage of vertical involvement of the epithelium by the neoplastic melanocytes being contentious (despite this having been defined by the fifth AJCC/TNM system.^12^ In 2022, the WHO-EYE05 editorial board modified the WHO-EYE04 C-MIL system to include benign melanosis, not only the malignant preinvasive lesions. It also proposed “subsuming” MIS into the category “high-grade C-MIL,” thereby dissolving the arbitrary “line” between those lesions with severe melanocytic atypia and MIS (Table 1).

In the current study, when using the modified WHO-EYE04 C-MIL system, the interobserver kappa outcomes between pathologists showed that 5 observers produced fair agreement with the consensus, 8 with moderate agreement, and only one with substantial agreement. In contrast, with the WHO-EYE05 3-tiered C-MIL system, greater consensus among pathologists was Achieved—ie, only one observer showed fair agreement, 6 showed moderate agreement, and 7 showed substantial agreement. Hence, greater reproducibility between pathologists can be obtained with a 3-tiered C-MIL scoring system—ie, “3 is company, and 4 is a crowd.”

Despite these encouraging results, this study has revealed that certain grades of C-MIL pose classification challenges for even expert eye pathologists. In both the 4-tiered WHO-EYE04 and the 3-tiered WHO-EYE05 systems, the consensus grade of benign melanosis showed the highest agreement at 74.5%, with the main differential grade being low-grade C-MIL (Table 3). This indicates that most pathologists are comfortable with applying the criteria for benign melanosis in Table 1. However, in ~15% of benign melanosis cases, there is an indication that discriminating foci of benign hyperplastic melanocytes in the mildly atypical melanocytes of low-grade C-MIL is challenging. Some pathologists may “err on the side of caution” and be overcalling a benign lesion as low-grade C-MIL to perhaps allow patient follow-up, rather than having them being discharged from the clinic.

For the high-grade C-MIL using the 4-tiered modified WHO-EYE04 C-MIL system, the agreement between pathologists was only 55.5%, with the nonagreeing opinions being roughly equally split between low-grade C-MIL and “MIS.” Calling a high-grade melanocytic lesion “MIS” would not necessarily pose significant issues, given that this study, and several others previously,^1–4,6,12–16^ have shown that high-grade C-MIL and MIS recur and are associated with invasive melanoma and therefore merit being merged as high-grade C-MIL (as per WHO-EYE05 C-MIL), with more favorable kappa statistics outcomes. However, calling a high-grade melanocytic lesion one of a significantly lower grade—eg, by interpretational down-grading of cytologic atypia and perhaps not recognizing the “significant” vertical spread of melanocytic cells—would lead to potential under treatment of the patient. The WHO-EYE05 C-MIL proposal with high-grade C-MILs including MIS led to an improved agreement between of pathologists of 85.4%. Low-grade C-MIL still represented the main “nonagreeing grade,” a challenge that must be addressed in future studies, perhaps with the aid of digital pathology and the application of machine learning. In Table 5, we have summarized possible causes of low consensus levels in the low-grade C-MIL and how they could be overcome in future studies.

Regarding the use of IHC to assist pathologists in grading C-MILs, Milman et al noted in their study that only a subset of cases had sufficient material for ancillary IHC, so that their use on grading classification was not possible.^5^ In this present study, although the impact of individual immunostains on C-MIL grading was not specifically studied, the responses from the participating pathologists indicated how morphology and IHC were clearly used in tandem to assess C-MIL grading. This is reflected in how different immunostains were employed for grading different C-MIL, ie, preference for SOX10 for benign melanosis and PRAME for helping in higher-grade lesion assessment, the latter being concordant with previous studies.^17–20^

At the 2 extreme ends of the classification (benign melanosis and MIS), it appears that IHC is employed to already confirm a strong morphologic suspicion, which is probably why these grades exhibit the highest agreement. For higher-grade lesions, there was use of 2 or more antibodies to likely confirm the interpretation. For low-grade C-MIL, IHC may be used more actively to classify uncertain morphology, with its inherent chance of over or under-calling a grade.

Pathologists assessing a conjunctival melanocytic intraepithelial lesion should work with a good-quality H&E section. Cases may require the assessment of at least 3 levels for a complete evaluation. Under the WHO-EYE05, it may also be desirable to employ at least one immunohistochemical stain to confirm melanocytic phenotype, facilitate C-MIL grading, and determine disease extent.^6^ The immunostains will depend on the pathologist’s preference and the laboratory’s experience, but markers could include Melan-A, SOX10, HMB45, MITF, and PRAME.^21^

This study has some limitations that may have confounded grading of the C-MILs. One particular limitation of the study was that it was undertaken on a digital platform. Although this affords the great advantage of sharing the same material among the participating pathologists across the globe (particularly during a pandemic!), the uptake of using digital pathology for routine reporting is highly variable among pathologists, with the mainstay still being the examination of glass slides under a light microscope.

In summary, this study supports the WHO-EYE05 system for grading C-MIL with its 3-tiered system of benign melanosis, low-grade C-MIL, and a high-grade C-MIL that includes conjunctival MIS. Our study highlights that there are continuing challenges with applying the system to low-grade C-MIL with poorer consensus between pathologists. It is anticipated that the application of machine learning and novel algorithms based on larger numbers of digitalized slides, potentially with associated genetic alterations, will assist in C-MIL grading in the future.

## Supplementary Material

Figure S1

Tables S1-S3

The online version contains supplementary material available at https://doi.org/10.1016/j.labinv.2023.100281

## Figures and Tables

**Table 1:** WHO 2022 classification of C-MIL in the WHO-EYE05

WHO	Acceptable alternative terminology	Increased cellularity	Histologic features	Risk of association with or progression to invasive melanoma
Not applicable	Benign melanosis c-MIN (grades (0–1) PAM without atypia	No/Minimal	Conjunctiva! Hypermelanosis (increased pigment in epithelial cells without melanocytic hyperplasia or atypia). Slight or focal melanocytic hyperplasia without atypia (parabasal melanocytes with condensed round nuclei, smaller than basal epithelial cell, inconspicuous nucleoli, and inconspicuous cytoplasm) may be seen.	None
Low-grade C-MIL	PAM with mild atypia c-MIN (grades 2–4)	Yes	Predominantly basilar melanocytic proliferation with low-grade atypia (dendritic or small-to-moderate size polyhedral, usually nonepithelioid melanocytes with round-to-irregular nuclear contours, often nuclear hyperchromasia, inconspicuous nucleoli, and inconspicuous or scant cytoplasm).	Lower
High-grade C-MIL	PAM with moderate to severe atypia c-MIN (grade 5–10)	Yes	More confluent basilar and significant nonbasilar proliferation of melanocytes with high-grade atypia (moderate to severe), evidence of intraepithelial nested and/or pagetoid growth, and epithelioid cell cytomorphology.	Higher
	Melanoma in situ	Yes	The term melanoma in situ may be used for (1) the most atypical high-grade C-MILs involving close-to-full thickness of the epithelium and (2) histologically obvious melanomas without documented evidence of subepithelial invasion.	Highest

C-MIL, conjunctival melanocytic intraepithelial lesion; PAM, primary acquired melanosis.

**Table 2:** Kappa statistics with 95% CI for the 14 observers compared with the consensus diagnosis in the 3-tiered system

Observer	Kappa	Lower 95% CI	Upper 95% CI	Kappa for 3-tiered system significantly higher than for 4-tiered system?
1	0.774	0.627	0.920	Yes
2	0.358	0.136	0.480	No
3	0.714	0.574	0.854	Yes
4	0.444	0.316	0.573	Yes
5	0.593	0.446	0.741	Yes
6	0.604	0.466	0.743	Yes
7	0.544	0.412	0.677	No
8	0.601	0.463	0.739	No
9	0.651	0.510	0.792	Yes
10	0.586	0.452	0.719	Yes
11	0.670	0.525	0.815	Yes
12	0.673	0.524	0.823	Yes
13	0.590	0.448	0.732	Yes
14	0.427	0.296	0.559	No

**Table 3:** Comparison of C-MIL agreements between the consensus and participating pathologists

Consensus grades (N = 4	Participating pathologists’ responses (N = 14)					
	Agreed with consensus	Disagreement with consensus	Total responses	% Agreement	% Disagreement	Breakdown of non-consensus grades
Benign (*n* = 28)	290	101	391	74.5	25.5	91 LG C-MIL; 10 HG C-MIL
LG C-MIL (*n* = 13)	70	111	181	38.7	61.3	40 benign; 67 HG C-MIL; 4 in situ
HG C-MIL (*n* = 37)	287	230	517	55.5	44.5	18 benign; 100 LG C-MIL; 112 in situ
MIS (*n* = 27)	240	136	376	63.8	36.2	4 benign; 8 LG C-MIL; 124 HG C-MIL
HG C-MILc (*n* = 64)	763	130	893	85.4	14.6	22 benign; 108 LG C-MIL

Benign, benign melanosis; HG C-MIL, high-grade conjunctival melanocytic intraepithelial lesion; HG C-MILc, high-grade conjunctival melanocytic intraepithelial lesion and MIS combined; LG C-MIL, low-grade conjunctival melanocytic intraepithelial lesion; MIS, melanoma in situ.

**Table 4:** Outcome of developing local recurrence and developing invasive melanoma (MM) with the 3-tiered system

Consensus grade	No. not developing recurrence	No. developing recurrence	% developing recurrence	No. not developing MM	No. developing MM	% developing MM
Benign	28	0	0	28	0	0
LG C-MIL	12	1	8	13	0	0
HG C-MILc	42	22	34	34	30	47

Benign, benign melanosis; HG C-MILc, high-grade conjunctival melanocytic intraepithelial lesion and MIS combined; LG C-MIL, low-grade conjunctival melanocytic intraepithelial lesion.

**Table 5:** Tabular summary of possible sources of discrepancy between pathologists in the assessment of the low-grade C-MIL

Possible causes of low-agreement levels for low-grade C-MIL	Possible solutions
Data-based	
Lack of availability of complete clinical data for appropriate clinical-pathologic correlation	This is essential in low-grade lesion interpretation. Inclusion of all details and clinical images where possible
Technology-based	
Unfamiliarity with digital platforms	Greater exposure to digital pathology interpretation
Morphologically-based	
Variation of H&E staining from other institutions	Repeat analysis of cases using both digital and slides
Preferred reliance on H&E sections only	Increased exposure to IHC results of C-MIL
Varying thickness of conjunctival epithelium between cases may have influenced each pathologist’s assessment of grade of C-MIL	Application of artificial intelligence (AI) to digitized slides may provide more accurate measurements of vertical involvement by atypical melanocytes
Immunohistochemistry-based	
Unfamiliarity with all IHC stains	Increased exposure to IHC results of C-MIL
Differing intensity of IHC stains, reflecting the “real world” variation between and within laboratories	Perform of all IHC in one center for future studies
Pathologist-based and Medical-system based	
“Erring on the side of caution”	This is difficult to cater for, and varies between cases and centers (clinician thresholds), given that each patient’s situation differs
Litigation-based	
Medicolegal risks vary considerably between countries, and influence pathologists’ use/not use of additional tests, as well as their interpretation of C-MIL.	Difficult to cater for: implementation of AI may enable test flow pathways, and the interpretation of stains

C-MIL, conjunctival melanocytic intraepithelial lesion; H&E, hematoxylin and eosin; IHC, immunohistochemistry.

## Data Availability

All data generated and analysed during this study are included in this published article.
