# Supplementary material for: A Multicenter Study Validates the WHO 2022 Classification for Conjunctival Melanocytic Intraepithelial Lesions With Clinical and Prognostic Relevance
Source: Lab Invest. Author manuscript; Available in PMC 2025 Aug 4. (PMC12320950; doi:10.1016/j.labinv.2023.100281)
Supplement: Figure S1 [file NIHMS2094283-supplement-Figure_S1.pptx]

## Slide 1
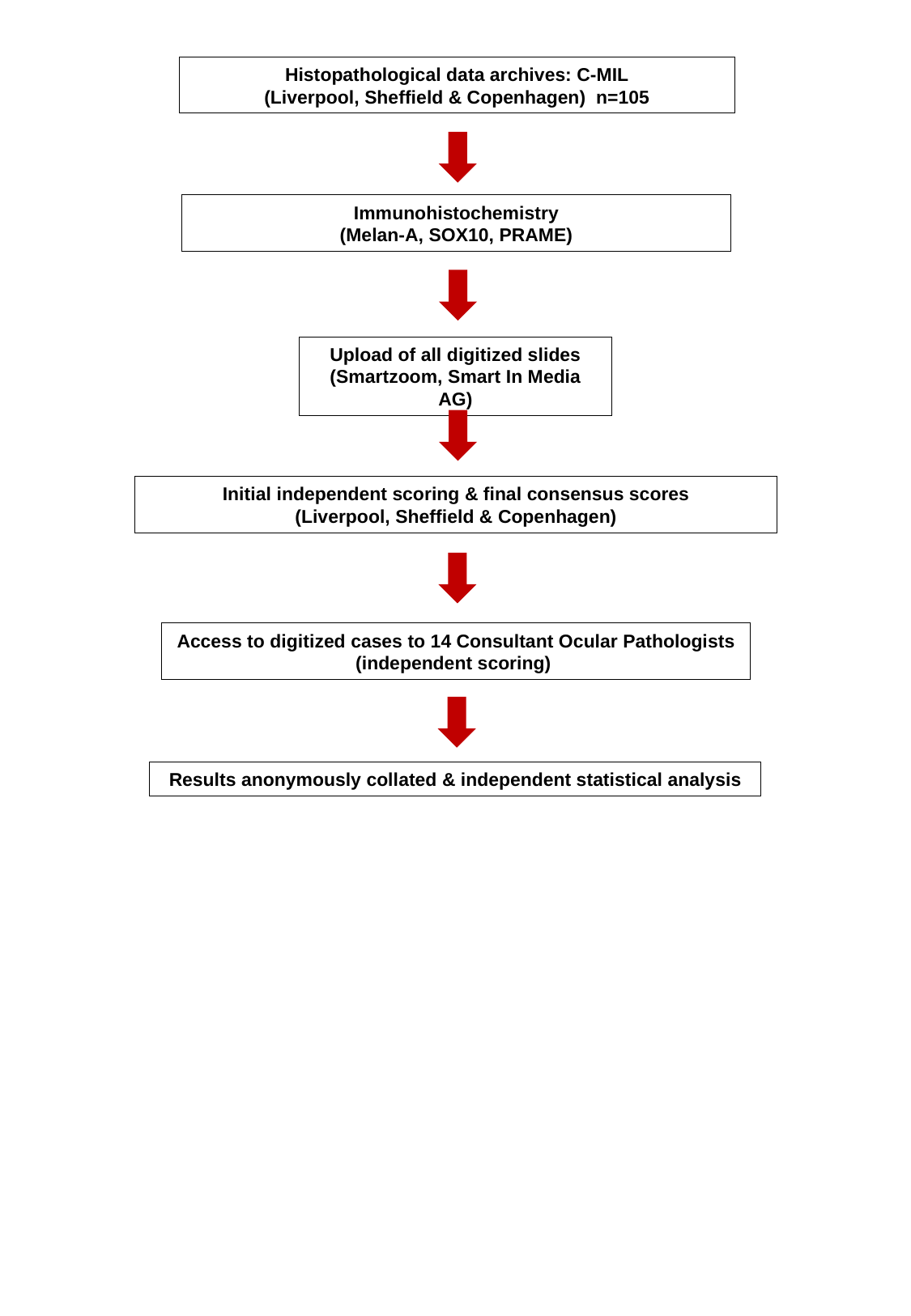

Histopathological data archives: C-MIL
(Liverpool, Sheffield & Copenhagen) n=105
Immunohistochemistry
(Melan-A, SOX10, PRAME)
Upload of all digitized slides (Smartzoom, Smart In Media AG)
Initial independent scoring & final consensus scores
(Liverpool, Sheffield & Copenhagen)
Access to digitized cases to 14 Consultant Ocular Pathologists (independent scoring)
Results anonymously collated & independent statistical analysis
