# Supplementary material for: A Multicenter Study Validates the WHO 2022 Classification for Conjunctival Melanocytic Intraepithelial Lesions With Clinical and Prognostic Relevance
Source: Lab Invest. Author manuscript; Available in PMC 2025 Aug 4. (PMC12320950; doi:10.1016/j.labinv.2023.100281)
Supplement: Tables S1-S3 [file NIHMS2094283-supplement-Tables_S1-S3.docx]

**Supplementary Table 1**: Kappa statistics with 95% confidence intervals for the 14 observers compared with the consensus diagnosis in the 4-tiered system.

| Observer | Kappa | Lower 95% CI | Upper 95% CI |
| --- | --- | --- | --- |
| 1 | 0.643 | 0.528 | 0.758 |
| 2 | 0.306 | 0.201 | 0.411 |
| 3 | 0.442 | 0.340 | 0.543 |
| 4 | 0.334 | 0.231 | 0.437 |
| 5 | 0.469 | 0.354 | 0.584 |
| 6 | 0.445 | 0.335 | 0.556 |
| 7 | 0.495 | 0.390 | 0.601 |
| 8 | 0.534 | 0.423 | 0.644 |
| 9 | 0.532 | 0.420 | 0.644 |
| 10 | 0.408 | 0.230 | 0.516 |
| 11 | 0.532 | 0.419 | 0.645 |
| 12 | 0.317 | 0.224 | 0.410 |
| 13 | 0.449 | 0.341 | 0.556 |
| 14 | 0.342 | 0.233 | 0.451 |

**Supplementary Table 2**: Outcome of developing local recurrence and developing invasive melanoma with the 4-tiered system. Benign=benign melanosis; LG C-MIL=Low-grade conjunctival melanocytic intraepithelial lesion; HG C-MIL=High-grade conjunctival melanocytic intraepithelial lesion; MIS=melanoma in situ.

| Consensus grade | No. not developing recurrence | No. developing recurrence | % developing recurrence | No. not developing Melanoma | No. developing Melanoma | % developing Melanoma |
| --- | --- | --- | --- | --- | --- | --- |
| **Benign** | 28 | 0 | 0 | 28 | 0 | 0 |
| **LG C-MIL** | 12 | 1 | 8 | 13 | 0 | 0 |
| **HG C-MIL** | 30 | 7 | 19 | 27 | 10 | 27 |
| **MIS** | 12 | 15 | 55 | 7 | 20 | 74 |

**Supplementary Table 3a**. Preferred immunostain(s) amongst the participating pathologists for the various C-MILs where there was agreement with the consensus grade. Benign=benign melanosis; LG C-MIL=Low-grade conjunctival melanocytic intraepithelial lesion; HG C-MIL=High-grade conjunctival melanocytic intraepithelial lesion; MIS=melanoma in situ; HG C-MILc=High-grade conjunctival melanocytic intraepithelial lesion and MIS combined.

| Consensus grade | SOX10 | Melan-A | PRAME | Melan-A  SOX10 | Melan-A PRAME | SOX10 PRAME | Melan-A  SOX10  PRAME | Total responses |
| --- | --- | --- | --- | --- | --- | --- | --- | --- |
| **Benign** | 112 (45%) | 72 (29%) | 0  (0%) | 44  (18%) | 6  (2%) | 5  (2%) | 9  (4%) | 248 |
| **LG C-MIL** | 15  (24%) | 23  (37%) | 4  (6%) | 14  (23%) | 1  (2%) | 2  (3%) | 3  (5%) | 62 |
| **HG C-MIL** | 47  (19%) | 108  (44%) | 14  (6%) | 44  (18%) | 9  (4%) | 12  (5%) | 11  (4%) | 245 |
| **MIS** | 32  (15%) | 45  (21%) | 19  (9%) | 54  (25%) | 16  (8%) | 5  (2%) | 42  (20%) | 213 |
| **HG C-MILc** | 79  (17%) | 153  (34%) | 33  (7%) | 98  (21%) | 25  (5%) | 17  (4%) | 53  (12%) | 458 |

**Supplementary Table 3b**. Preferred immunostain(s) amongst the participating pathologists for the various C-MILs where there was no agreement with the consensus grade. Benign=benign melanosis; LG C-MIL=Low-grade conjunctival melanocytic intraepithelial lesion; HG C-MIL=High-grade conjunctival melanocytic intraepithelial lesion; MIS=melanoma in situ; HG C-MILc=High-grade conjunctival melanocytic intraepithelial lesion and MIS combined.

| Consensus grade | Participating pathologist grade at variance to consensus | SOX10 | Melan-A | PRAME | Melan-A  SOX10 | Melan-A  PRAME | SOX10 PRAME | Melan-A SOX10 PRAME | Total responses |
| --- | --- | --- | --- | --- | --- | --- | --- | --- | --- |
| Benign melanosis | LG C-MIL | 5  (7%) | 46  (61%) | 0  (0%) | 18  (24%) | 4  (5%) | 0  (0%) | 2  (3%) | 75 |
|  | HG C-MIL | 0  (0%) | 4  (66%) | 0  (0%) | 1  (17%) | 1  (17%) | 0  (0%) | 0  (0%) | 6 |
| LG C-MIL | Benign melanosis | 19  (56%) | 6  (18%) | 0  (0%) | 6  (18%) | 1  (3%) | 2  (7%) | 0  (0%) | 34 |
|  | HG C-MIL | 8  (14%) | 25  (44%) | 5  (9%) | 11  (19%) | 3  (5%) | 4  (7%) | 1  (2%) | 57 |
|  | In-situ | 0  (0%) | 0  (0%) | 0  (0%) | 2  (2%) | 0  (0%) | 0  (0%) | 0  (0%) | 2 |
| HG C-MIL | Benign melanosis | 7  (47%) | 2  (13%) | 2  (13%) | 1  (7%) | 1  (7%) | 2  (13%) | 0  (0%) | 15 |
|  | LG C-MIL | 28  (34%) | 22  (27%) | 6  (7%) | 13  (16%) | 1  (1%) | 6  (7%) | 7  (8%) | 83 |
|  | In-situ | 8  (10%) | 18  (21%) | 3  (4%) | 33  (39%) | 10  (12%) | 2  (2%) | 10  (12%) | 84 |
| MIS | Benign melanosis | 0  (0%) | 1  (25%) | 0  (0%) | 0  (0%) | 1  (25%) | 0  (0%) | 2  (50%) | 4 |
|  | LG C-MIL | 2  (29%) | 3  (43%) | 1  (14%) | 0  (0%) | 0  (0%) | 1  (14%) | 0  (0%) | 7 |
|  | HG C-MIL | 23  (24%) | 20  (20%) | 9  (9%) | 9  (9%) | 10  (10%) | 5  (5%) | 21  (22%) | 97 |
| HG C-MILc | Benign melanosis | 7  (37%) | 3  (16%) | 2  (11%) | 1  (4%) | 2  (11%) | 2  (11%) | 2  (11%) | 19 |
|  | LG C-MIL | 30  (33%) | 25  (28%) | 7  (8%) | 13  (14%) | 1  (1%) | 7  (8%) | 7  (8%) | 90 |
